# Dr. William Jenner

**Published:** 1861-05

**Authors:** 


DR. WILLIAM JENNER, Physician to University College Hospital, and the author of several works on fever, has been appointed Physician Extraordinary to the Queen, in place of Dr. William Baly, who recently met with so sad an end.

